# Multiplex immunofluorescence microscopy assays for pharmacodynamic assessment of MET tyrosine kinase activation in the plasma membrane and nucleus

**DOI:** 10.1371/journal.pone.0349090

**Published:** 2026-05-12

**Authors:** Tony Navas, Apurva K. Srivastava, Scott M. Lawrence, Melinda G. Hollingshead, Sergio Y. Alcoser, Sarah B. Miller, Robert J. Kinders, Donald P. Bottaro, W. Marston Linehan, Shivaani Kummar, James H. Doroshow, Ralph E. Parchment

**Affiliations:** 1 Clinical Pharmacodynamic Biomarkers Program, Applied/Developmental Research Directorate, Frederick National Laboratory for Cancer Research, Frederick, Maryland, United States of America; 2 Biological Testing Branch, Developmental Therapeutics Program, National Cancer Institute at Frederick, Frederick, Maryland, United States of America; 3 Division of Cancer Treatment and Diagnosis, National Cancer Institute, Bethesda, Maryland, United States of America; 4 Urologic Oncology Branch, Center for Cancer Research, National Cancer Institute, Bethesda, Maryland, United States of America; 5 Center for Cancer Research, National Cancer Institute, Bethesda, Maryland, United States of America; Weill Cornell Medicine, UNITED STATES OF AMERICA

## Abstract

The HGF/SF (hepatocyte growth factor/scatter factor) receptor tyrosine kinase MET is overexpressed and/or activated in many tumors, providing therapeutic targets for antibody-drug conjugates and tyrosine kinase inhibitors. Reliable measurement of activated MET is fundamental for pharmacodynamic assessment of MET-targeted therapies and for expanded and proper use of such therapies in patients with tumors driven by activated MET with or without associated *MET* amplification or known activating mutations. To address the paucity of tools for directly measuring MET activation in tumor cells within patient biopsy specimens, we developed a robust, quantitative immunofluorescence microscopy assay to measure levels of pY^1235^MET and total MET in *in vitro*, *in vivo*, and patient tumor specimens. We validated this assay through assessment of MET inhibitor–treated preclinical models, peptide blocking experiments to demonstrate specificity, and concordance with corresponding measurements from the same specimens using a previously validated sandwich immunoassay of tumor lysates. Given the importance of plasma membrane-associated MET in initiating its canonical signaling cascades, as well as the demonstrated non-canonical signaling from nuclear localized MET in different tumor cell types and in response to various environmental stimuli, we developed assay capability to measure levels of pY^1235^MET and total MET within the plasma membrane or nucleus; these assays enable future explorations of the biological and clinical relevance of MET subcellular localization patterns. Finally, using tissue microarrays of over 50 resected tumor specimens from patients with colorectal carcinoma or non-small cell lung cancer, we demonstrated that tumor levels of pY^1235^MET do not always track total MET expression, suggesting that measurement of activated MET in tumor could hold potential as an independent biomarker to identify additional patients who might benefit from MET-directed targeted therapy—beyond those with tumor *MET* amplification, MET overexpression, or established MET-activating mutations.

## Introduction

Specific molecular aberrations resulting in hyperactivation of the receptor tyrosine kinase MET have been shown to be associated with poor prognosis and, in some cases, have been demonstrated to confer response to MET-targeted therapies in patients with solid tumors of various histologies, including non-small cell lung cancer (NSCLC), gastric cancer, colorectal carcinoma (CRC), and type 1 papillary renal cell carcinoma [1–4]. These aberrations include *MET* exon 14–skipping mutations that lead to alternatively spliced isoforms lacking a critical regulatory portion of the juxtamembrane domain, as well as *MET* gene amplification and/or protein overexpression [5]. To date, several clinical trials of MET-targeting agents have utilized *MET* amplification and/or exon 14–skipping mutations to select patients; while tumor exon 14–skipping mutations, which are known to yield constitutively activated MET, have been shown to confer sensitivity to MET inhibitors [1,2,5], *MET* gene amplification and even MET protein overexpression have often been insufficient for predicting response to MET-targeting agents [2,6,7], and the full repertoire of molecular defects that may confer sensitivity to MET inhibitors remains unestablished.

The identification and confirmation of additional predictive MET-activating mutations, as well as new therapeutic agents designed to inhibit MET signaling, can be facilitated by pharmacodynamic studies with assays that generate accurate and precise measurements of MET activation. Following hepatocyte growth factor (HGF) ligand binding and receptor dimerization, MET signaling is initiated by the autophosphorylation of tyrosine residues 1234 and 1235 (pY^1234/1235^) within the intracellular MET kinase domain; this, in turn, leads to the activating phosphorylation of tyrosine residues 1349 and 1356 in the MET C-terminus, to which adapter and signaling proteins are then recruited to mediate downstream signaling events [2]. Given the critical role of MET intracellular domain phosphorylation in the signal transduction process, several studies have attempted to examine phospho-MET expression levels in clinical tumor specimens; these include qualitative, immunohistochemistry (IHC)–based assessments of phospho-MET expression using anti–pY^1349^MET monoclonal [8] and anti–pY^1235^MET polyclonal antibodies [9]—which demonstrated no association between phospho-MET levels and patient survival—as well as a quantitative IHC assay using an anti–pY^1234/1235^MET monoclonal antibody [10]. However, in addition to the inherent limitations of qualitative analyses and the antibody specificity issues that have confounded prior quantitative microscopy-based assessments [11], many such prior attempts to assess phospho-MET levels have been complicated by the use of sample collection procedures that fail to properly mitigate the known instability of MET phospho-isoforms during even relatively short periods of ischemia [6]. To address the above issues, we previously developed, validated, and demonstrated clinical fitness-for-purpose of a chemiluminescence-based sandwich immunoassay—and companion procedures for specimen collection and processing that minimize ischemia time and stabilize labile post-translational protein modifications—to reliably measure full-length pY^1234/1235^MET in lysates of core needle tumor biopsy specimens [6,11,12]. This enzyme-linked immunosorbent assay (ELISA) also provides specific, quantitative measurements of full-length activated MET in patient tumor specimens [11,12]. However, because the pY^1234/1235^MET ELISA utilizes a capture antibody that recognizes the MET N-terminus to ensure quantitation of full-length MET specifically, this assay does not detect the biologically active, Y^1234/1235^–phosphorylated, intracellular C-terminal fragments resulting from proteolytic cleavage during MET signal transduction. In addition, the clinical feasibility of using such lysate-based assays as companion diagnostics for MET-targeting agents is limited by the requirement for a full-size 18-gauge core tumor biopsy specimen of sufficiently high viable tumor content—approximately ≥ 25% [13].

Lysate-based assessments of tumor pY^1234/1235^MET levels are also limited by the lack of subcellular localization information, which may hamper thorough delineation of the cellular response to MET inhibitors in clinical and preclinical mechanism-of-action studies. The intracellular trafficking of receptor tyrosine kinases is well documented [14], and several studies have demonstrated MET translocation to the nucleus in response to specific environmental stimuli, including changes in cell density [15], pH [16], oxidative stress [17], and HGF stimulation [8,18–20], though HGF-independent nuclear localization of MET has also been detected in some cancer cell lines [21–23]. MET contains a nuclear localization signal (NLS) located in the exon 14–encoded juxtamembrane region [16], and both the nuclear translocation of proteolytically cleaved MET C-terminal domain [15,16,21,23] and localization of full-length MET to the nuclear envelope [8,17,24] have been observed in cancer cell lines. Several biological roles have been described for nuclear MET, including phosphorylation of PARP1, which results in diminished response to PARP inhibitors [17]; upregulation of SOX and β‑catenin expression to promote epithelial-to-mesenchymal transition (EMT) [25]; and activation of NF-κB signaling via the upregulation of TAK1 expression [22]. The prognostic or predictive value of MET distribution in specific subcellular locations and/or phosphorylation states remains a matter of debate and may be dependent on tumor histology. Prior studies have found that plasma membrane–localized total MET—but not pY^1349^MET—is a prognostic factor in mesothelioma [8], that nuclear MET levels are associated with late-stage disease and poor overall survival in patients with hepatocellular carcinoma [22], and that neither cytoplasmic nor nuclear MET levels are prognostic in patients with oral squamous cell carcinomas [23].

To enable robust quantitation of activated MET at both the cellular and subcellular levels, including full-length MET and the C-terminal cleavage products resulting from proteolysis during signal transduction, we developed an immunofluorescence microscopy–based assay to measure MET and pY^1235^MET in patient tumor specimens. Because phosphorylation of Y^1234^ has been shown to be dispensable for MET activation under some circumstances—for example, in the presence of specific oncogenic MET kinase domain mutations—we aimed to instead quantitate levels of pY^1235^MET, which is universally required for MET signal transduction [26]. As detailed below, we used our novel monoclonal antibody (clone 23111) that, as we have shown previously [11], selectively recognizes Y^1235^-phosphorylated MET independently of Y^1234^ phosphorylation status for reliable measurement of pY^1235^MET tumor cell content, tumor cell nuclear content, and tumor cell plasma membrane content to enable detailed analyses of the temporal and spatial dynamics of MET activation and response to MET–targeted therapies.

## Materials and methods

### Antibodies for immunofluorescence analysis

The mouse monoclonal antibody against total MET (generated via immunization with a peptide corresponding to the MET C-terminus) was purchased from Cell Signaling Technology (CST; clone D1C2) and directly conjugated to Alexa Fluor (AF) 488; the AF647 or AF546-conjugated mouse monoclonal antibody MET4 (clone 8G6) [27,28] was used to measure the ectodomain of MET in formalin-fixed specimens of untreated cell lines, SNU-5 xenograft models treated with the MET/ALK tyrosine kinase inhibitor (TKI) crizotinib, and *hHGF* knock-in H596 models against total MET. A rabbit monoclonal antibody specific to pY^1235^MET (“anti‑pY^1235^MET”; clone 23111; US11340219B2, EP3430051B1), and with undetectable reactivity to the adjacent pY^1234^ site in MET, was previously discovered and developed in collaboration with Epitomics Inc. (Abcam) using phosphopeptide antigens corresponding to the MET amino acid sequences flanking residue 1235 [11]; this antibody is available via the Developmental Studies Hybridoma Bank (antibody registry ID: AB_2888958; https://dshb.biology.uiowa.edu/CPTC-MET-1). Clone 23111 was directly conjugated to AF546. The rabbit monoclonal antibody directed against a proprietary intracellular epitope of the alpha-1 subunit of Na+/K^+^—ATPase was purchased from Abcam (clone EP1845Y) and directly conjugated to AF647. The goat anti-rabbit AF546 secondary antibody was purchased from Life Technologies.

### Immunofluorescence staining of pY^1235^MET in HGF- and crizotinib-treated A549 and HT29 cells

A549 or HT29 cells were grown on chamber slides to 80% confluency before being serum starved for 24 hours. Cells were incubated with or without 100 nM crizotinib (a MET/ALK TKI) for 4 hours prior to stimulation with 20 ng/ml recombinant HGF (HEK293-derived, Peprotech)—the MET ligand—for 15 min (A549) or 24 hours (HT29) in serum-free media. Cells were fixed in 10% NBF for 15 minutes, permeabilized in 1% BSA in PBS with 0.5% Tween-20 and 0.1% Triton X-100, and stained with anti‑pY^1235^MET antibody (10 μg/mL) followed by 10 μg/mL anti-rabbit AF546 secondary antibody. Slides were imaged using a Nikon 90i Andor Camera, and the images were analyzed using NIS Elements Software.

### Analysis of total MET, pY^1235^MET, and pY^1235^MET/total MET ratios in untreated cancer cell lines

Formalin-fixed, paraffin-embedded (FFPE) cell pellets harvested from untreated GTL-16, HT29, MDA-MB-231, and SNU-1 cell lines (NCI Developmental Therapeutics Program Tumor Repository) were stained with DAPI and fluorescence-conjugated anti-MET [MET4 [27]] and anti-pY^1235^MET antibodies. Quantitation of total MET and pY^1235^MET staining was performed using Definiens Tissue Studio software.

### Immunofluorescence and Western blot analysis of peptide blocking experiments in GTL-16 xenograft models or crizotinib-treated GTL-16 and A549 cells

For immunofluorescence microscopy analysis, untreated flash-frozen GTL-16 xenograft tumor tissues or cell culture pellets were incubated with fluorescence-conjugated monoclonal anti-MET and anti-pY^1235^MET antibodies that were preincubated overnight at 4°C with either buffer, an unphosphorylated MET peptide corresponding to amino acids 1229–1240 (MYDKEYYSVHNK), or the same peptide phosphorylated at tyrosine 1234 (MYDKE[pY]YSVHNK), at tyrosine 1235 (MYDKEY[pY]SVHNK), or at both of these tyrosine residues (MYDKE[pY][pY]SVHNK). Each peptide was present at 20 times the molar concentration of the anti-pY^1235^MET antibody.

For Western blotting analysis of *in vitro* peptide blocking experiments, GTL-16 cells were first treated with crizotinib (0, 25, or 100 nM) for 4 hours. Cell pellets were harvested, lysed in Cell Extraction Buffer (Invitrogen) containing PhosSTOP Phosphatase Inhibitor Cocktail (Roche) and cOmplete Mini EDTA-free Protease Inhibitor Cocktail tablets (Roche), separated by electrophoresis on a NuPAGE Novex 4–12% Bis-Tris Protein Gel (Invitrogen), and transferred to an iBlot Transfer Stack nitrocellulose membrane (Invitrogen). Tyrosine 1235–phosphorylated MET was detected via digoxigenin (DIG)-conjugated rabbit anti-pY^1235^MET primary antibody and an AF790-conjugated anti-DIG secondary antibody (Licor), while full-length MET was assessed using the unconjugated anti–total MET D1C2 antibody followed by a 680-LT–conjugated anti-rabbit secondary antibody. Each blot was incubated overnight with the antibodies as well as either buffer or one of the 4 aforementioned MET peptides (unphosphorylated, pY^1234^, pY^1235^, or pY^1234/1235^) present at a molar concentration 30 times the molar concentration of the anti-pY^1235^MET primary antibody. Following 3 rinses with Licor buffer, blots were visualized using Odyssey software.

### Animal ethics statement

Frederick National Laboratory is accredited by the Association for Assessment and Accreditation of Laboratory Animal Care International and follows the Public Health Service Policy for the Care and Use of Laboratory Animals. All the studies were approved by the NCI Institutional Animal Care and Use Committee (IACUC) and conducted according to an approved animal care and use committee protocol in accordance with the procedures outlined in the “Guide for Care and Use of Laboratory Animals” (National Research Council; 1996; National Academy Press; Washington, DC).

### Animal model experiments

Athymic nude mice (nu/nu NCr; NCI Biological Testing Branch in-house breeding colonies, FNLCR) were implanted with the specified human cancer cell lines by subcutaneous implantation. For the experiment with H596 xenograft tumors, H596 cells were implanted into either wildtype SCID or homozygous human *HGF* knock-in (*hHGF*^*ki/ki*^) mice as described previously [29]; tumors were harvested after 33 days. The animal use program at the FNLCR is accredited by Association for Assessment and Accreditation of Laboratory Animal Care International and follows the USPHS Policy for the Care and Use of Laboratory Animals. All animal studies were conducted in compliance with an approved animal care and use committee protocol.

Mice were housed in sterile, filter-capped, polycarbonate cages maintained in a barrier facility on a 12-hour light/dark cycle and were provided sterilized food and water ad libitum. For all experiments, all mice were sacrificed by IACUC-approved methods (consistent with AVMA Guidelines for Euthanasia), including induction and maintenance of isoflurane anesthesia with exsanguination or exposure to carbon dioxide for a minimum of 10 minutes followed by additional observation or cervical dislocation. All animals were monitored daily for clinical signs of distress (hunched posture, roughened haircoat, inactivity, general body condition, weight loss, inappetence, and/or self-isolation). Animals showing signs of distress were treated with wet feed/Hydrogel^TM^ if signs were mild (weight < 20%; only 1 clinical sign apparent) or humanely euthanized on that day, as described above, if signs were more than mild.

Crizotinib (PF 02341066; NSC D749769-Y), tivantinib (NSC 758242), and pazopanib (NSC 737754) were obtained from the Developmental Therapeutics Program (DTP), NCI. Crizotinib and pazopanib were administered by oral gavage in a saline vehicle. Tivantinib was administered orally for 8 days in a PEG 400:20% vitamin E TPGS solution (60:40) vehicle. All drug-treated xenograft tumor specimens were collected as part of a prior study using methods to preserve labile protein phosphorylation sites [11]. Tumor biopsy specimens were collected using 18-gauge Temno Evolution soft tissue biopsy devices (Merit Medical System), while whole-xenograft tumors were collected by standard excision technique and immediately sectioned into 2–4 equal pieces with fine-point scissors. Each biopsy specimen or tumor quadrant was immediately flash-frozen in an O-ring–sealed, conical-bottom, screw-capped 1.5‑mL Sarstedt cryovial (pre-cooled in liquid nitrogen) by touching the biopsy to the inside of the tube within one minute of completing the collection procedure, and stored at ‑80°C. At the time of analysis of core needle biopsies, specimens were thawed in room temperature, 10% neutral buffered formalin (NBF) and processed into paraffin blocks with biomarker-positive control tissue for sectioning, using a previously described method for preserving phosphoproteins and other labile biomarkers [11]. At the time of analysis of tumor quadrants, specimens were sliced (inside cryovial tubes) into small, needle biopsy–sized sections and immediately fixed in room temperature, 10% neutral buffered formalin (NBF) containing PhosSTOP phosphatase inhibitor cocktail (Roche) for 24 hours, then embedded in paraffin for sectioning.

### Human research ethics statement

Clinical studies NCT01468922 and NCT00026884 were approved by the National Cancer Institute Institutional Review Board. All patients gave written informed consent for study participation. Study design and conduct complied with all applicable regulations, guidances, and local policies.

### Human tumor biopsies and tissue microarrays

Core needle (18-gauge) biopsies of metastatic lesions were collected from patients with advanced, refractory cancers enrolled in clinical trials conducted by the NCI Division of Cancer Treatment and Diagnosis (DCTD) and Center for Cancer Research at the NIH Clinical Center, Bethesda, Maryland: studies NCT01468922 and NCT00026884. Patients were accrued to study NCT01468922 from January 23, 2012 through March 18, 2015. For retrospective analyses of archived tumor tissue specimens collected under protocol NCT00026884, specimens were accessed for research purposes on October 12, 2011, and authors did not have access to identifiable patient information for these specimens. Each biopsy core was placed in a pre-chilled cryogenic vial, frozen within 2 minutes of collection, and stored at −80°C. At the time of analysis, biopsy specimens were thawed in fixative and paraffin-blocked with biomarker-positive control tissue for sectioning, using a previously described method for preserving phosphoproteins and other labile biomarkers [11]. A summary of the procedures for processing clinical and preclinical samples can be found in Standard Operating Procedures (SOP) 340507, 340550, 340546, and 340548 published on the NCI/DCTD website (http://dctd.cancer.gov/ResearchResources/ResearchResources-biomarkers.htm).

Tissue microarrays containing resected tumor tissue from patients with colorectal carcinoma (CRC) or NSCLC were acquired from Indivumed GmbH. Tissue specimens were subjected to ischemia times of < 5 minutes while transferred into neutral buffered formalin prior to blocking and preparation of each tissue microarray for sectioning.

### Crizotinib-treated SNU-5 tissue microarrays

Mice harboring SNU-5 xenograft tumors were treated once daily (QD) for 4 days via oral gavage (PO) of vehicle (water) or 12.5 or 25 mg/kg crizotinib (*n* = 4 animals per group), and three 18-gauge Temno Evolution biopsy cores were collected per animal 4 hours after administration of the final drug dose. Cores were placed into pre-chilled cryovials, which were transferred directly into liquid nitrogen and subsequently stored at −80°C. Upon retrieval from cryostorage, the samples were thawed under formalin fixative and then paraffin embedded. FFPE sections were stained with DAPI and fluorescence-conjugated anti-pY^1235^MET (10 μg/mL), and anti-MET (clone D1C2, 2.5 μg/mL) antibodies.

### IFA analysis of total MET and pY^1235^MET in xenograft and clinical specimens

Fluorescence microscopy images were collected using an Aperio FL Immunofluorescence Slide Scanner and Spectrum Analysis software (Leica Biosystems) or, for confocal images, using a Nikon Eclipse Ni-E microscope with an A1si scan head. Total MET, pY^1235^MET, and the total MET/pY^1235^MET ratio were determined as described above.

For analysis of plasma membrane–localized MET, Na^+^/K^+^-ATPase expression was used to segment the plasma membrane. Total MET and pY^1235^MET expression within the plasma membrane were then quantitated using Definiens Tissue Studio software and marker area analysis. Marker expression levels were independently assessed using isotype controls and biologically relevant cells lines to determine positive intensity thresholds. For analysis of nuclear-localized total MET and pY^1235^MET expression, DAPI-positive nuclear regions were segmented prior to measuring MET expression within the nuclei using Definiens Tissue Studio software and marker area analysis.

### Statistical analysis

*P*-values for comparisons of analyte levels between groups were derived from unpaired, nonparametric Mann-Whitney tests. Assessments of the association between ELISA and IFA values were performed using Spearman’s rank-order correlation analyses.

## Results

### Sensitivity and specificity of an immunofluorescence microscopy assay to quantify and localize Y^1235^-phosphorylated MET in tumor cells

**Fig 1:**
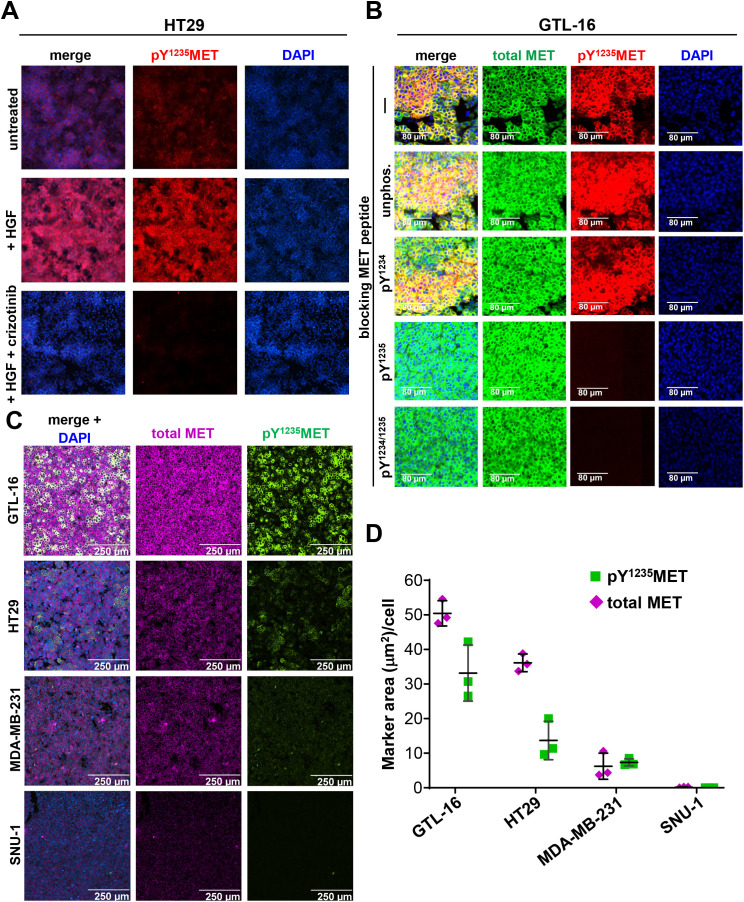
*In vitro* and *in vivo* validation of a multiplex immunofluorescence assay (IFA) for quantitation of pY^1235^MET. **A,** The pY^1235^MET IFA detects crizotinib-mediated inhibition of HGF-induced MET activation in HT29 colorectal adenocarcinoma cells. HT29 cells were incubated with or without 100 nM crizotinib for 4 hours prior to stimulation with 20 ng/mL HGF for 24 hours. Cells were harvested, fixed in 10% NBF for 10 minutes, and stained with DAPI, rabbit anti-pY^1235^MET antibody [clone 23111, [11]], and AF546-conjugated anti-rabbit secondary antibody. **B,** Specificity of anti-pY^1235^MET antibody IFA signal demonstrated by peptide blocking. FFPE sections of untreated *MET*-amplified GTL-16 gastric carcinoma xenograft tumors were incubated with an AF488-conjugated anti-MET antibody (clone D1C2, Cell Signaling Technology) or the anti-pY^1235^MET antibody together with either buffer (−), an unphosphorylated MET peptide corresponding to amino acids 1229−1240 (unphos.), or the same peptide phosphorylated at tyrosine 1234 (pY^1234^), at tyrosine 1235 (pY^1235^), or at both of these tyrosine residues (pY^1234/1235^). Each peptide was present at 30 times the molar concentration of the anti-pY^1235^MET antibody. **C, D,** Multiplex IFA-based quantitation of pY^1235^MET (green) and total MET (magenta) in cancer cell lines with a range of baseline MET expression and activation levels, including: GTL‑16, a *MET*-amplified cell line with constitutive, ligand-independent MET signaling [11]; HT29, a non-*MET*-amplified cell line with moderate baseline levels of MET expression and activation [31]; the MDA-MB-231 triple-negative breast cancer cell line, which exhibits moderate baseline MET expression and very weak baseline MET activation [32]; and the SNU-1 gastric carcinoma cell line, which has negligible MET expression [33]. Total MET was detected using the AF647-conjugated mouse monoclonal antibody MET4 (clone 8G6). **C,** Representative immunofluorescence microscopy images showing total MET and pY^1235^MET levels for the 4 cell lines. **D,** Quantitation of total MET and pY^1235^MET in IFA images. The tumor cell area that is positive for each marker within each image field is divided by the number of nuclei in the same field to generate a measure of the marker area positive per cell, and such values are averaged over several image fields to obtain the mean marker area per cell for each cell line model. Each point represents the value for an individual image field; horizontal lines indicate means ± standard deviations.

To verify that our novel anti-pY^1235^MET antibody detects MET receptor activation in intact tumor cells, HT29 colorectal adenocarcinoma and A549 lung carcinoma cells, which have low and moderate baseline levels of activated MET, respectively [11], were treated *in vitro* with HGF, with or without the addition of the MET/ALK TKI crizotinib. This antibody enabled visualization of pY^1235^MET upregulation following treatment with 20 ng/mL HGF for 4 hours, as well as near elimination of the pY^1235^MET signal when cells were pre-incubated with 100 nM crizotinib prior to HGF treatment (Fig 1A and Supplementary S1A Fig). Similarly, this antibody detected HGF-induced pY^1235^MET upregulation in the H596 NSCLC cell line, which harbors a *MET* exon 14–skipping mutation that impairs receptor degradation and prolongs MET signaling (Supplementary S1A Fig); crizotinib preincubation of H596 cells only partially reduced this HGF-mediated pY^1235^MET upregulation, consistent with the previously reported modest activity of crizotinib in this cell line [30].

To define the antigen specificity of this anti-pY^1235^MET antibody, we next performed peptide blocking experiments using tumor tissue from the *MET*-amplified, GTL-16 gastric carcinoma xenograft model, which exhibits high baseline levels of MET signaling [11]. The pY^1235^MET staining observed in untreated GTL-16 tumors was eliminated by pre-incubation of the anti-pY^1235^MET antibody with a 30-fold molar excess of pY^1235^-phosphorylated or pY^1234^/pY^1235^–dually phosphorylated peptides corresponding to amino acids 1229–1240 of the full-length MET protein, while pre-incubation of the antibody with the corresponding unphosphorylated or pY^1234^-phosphorylated peptides did not attenuate the fluorescence intensity of anti-pY^1235^MET antibody staining (Fig 1B). As expected, staining of total MET using the C-terminus–directed antibody was unaffected by pre-incubation with any of the peptides corresponding to amino acids 1229–1240 (Fig 1B). Comparable results were obtained when the peptide blocking experiments were repeated using Western blotting (Supplementary S1B Fig).

To evaluate the sensitivity and quantitative capacity of this pY^1235^MET assay, we assessed pY^1235^MET and total MET levels in cell lines exhibiting a range of baseline MET activation levels. As expected, the GTL-16 cell line had high levels of both total MET and pY^1235^MET, while HT29, a non-*MET*-amplified cell line, exhibited moderate levels of both [31], and the MDA‑MB‑231 triple-negative breast cancer cell line showed weak baseline MET expression and very weak baseline MET activation [32] (Fig 1C and D). The SNU-1 gastric carcinoma cell line, which is known to have negligible MET expression [33], did not yield appreciable expression of either total MET or pY^1235^MET when assessed by IFA. These relative differences in pY^1235^MET and total MET across the 4 cell lines were reflected in both the immunofluorescence microscopy images (Fig 1C) and in the quantitative image analysis results generated using Definiens Tissue Studio (Fig 1D). As shown in Fig 1D, there was consistent measurement of the endpoint across multiple microscopy fields within each cell line model.

### Orthogonal and fitness-for-purpose validation of the pY^1235^MET immunofluorescence assay

**Fig 2:**
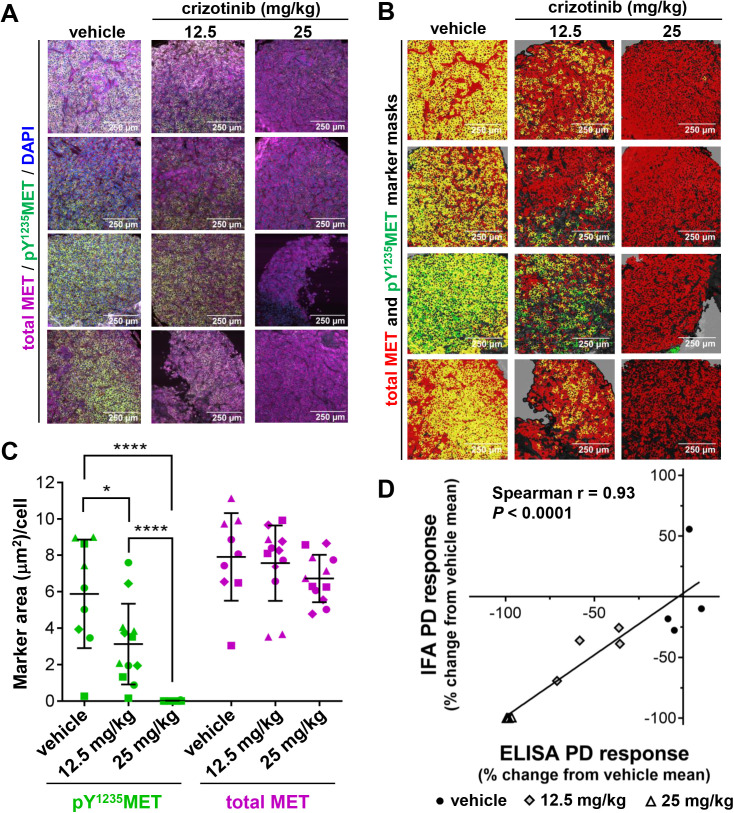
Fitness-for-purpose and orthogonal validation of IFA-based pY^1235^MET/total MET measurements in sections of FFPE tumor tissue from SNU-5 xenograft models treated with crizotinib. **A,** Tissue microarray core sections from SNU-5 xenograft models treated with vehicle or the indicated doses of crizotinib (QD × 4, PO); tumor cores were collected 4 hours following administration of the final dose and flash frozen. FFPE tissue sections were stained with DAPI, DIG-conjugated anti-pY^1235^MET antibody (clone 23111 [11]) followed by anti-DIG secondary antibody, and AF647-conjugated anti-MET antibody MET4 (clone 8G6) [27]. Images of tumor cores from each of the 4 animals in each treatment group are shown. **B,** Masked images of each tumor, showing total MET (red) and pY^1235^MET (green), for quantitative analysis using Definiens Tissue Studio. **C,** Quantitation of pY^1235^MET (green) and total MET (magenta) for IFA images shown above. Each point represents a measurement from a different area of a unique tumor core biopsy specimen; within each group, points with the same symbol indicate the cores harvested from the same animal. Horizontal lines indicate the mean and standard deviation for each group, and asterisks indicate significant differences between groups (****P < 0.0001; *n* = 4 animals and 1-3 cores/animal per group). **D,** Crizotinib-induced pharmacodynamic changes measured using a previously validated ELISA-based assay [11] provide orthogonal validation of IFA-based pY^1235^MET measurements. Each point represents the pharmacodynamic effect on MET activation as measured by IFA (pY^1235^MET) and ELISA (pY^1234/1235^MET/total MET ratio) for a single SNU-5 tumor from the indicated treatment group; measurements are shown as percent change from vehicle mean (i.e., 100*[sample value – vehicle mean]/vehicle mean). ELISA data were reported previously [11]. The Spearman correlation coefficient and corresponding *P*‑value are shown.

To validate the immunofluorescence microscopy-based assay for pY^1235^MET, we compared its performance to that of our previously developed ELISA-based assay that measures pY^1234/1235^MET and total, full-length MET in extracts of tumor specimens and reports their calculated ratio, “pY^1234/1235^MET/MET” [11]. This normalization of pY^1234/1235^MET to total MET levels is necessary in tumor lysate-based immunoassays to address inter-specimen variations in tumor content—an issue that is irrelevant for microscopy assays that use image segmentation approaches to restrict measurements to tumor cells. In SNU-5 xenograft models of *MET*-amplified gastric carcinoma treated with 12.5 or 25 mg/kg crizotinib daily for 4 days, IFA analysis of tumor cores collected 4 hours after administration of the final crizotinib dose revealed a significant, dose-dependent decrease in pY^1235^MET-positive area per cell, with no significant change in total MET levels (Fig 2A-C). The pharmacodynamic effects of crizotinib on MET activation levels as measured by the pY^1235^MET IFA were concordant with those measured using the previously validated pY^1234/1235^MET/MET ELISA with specimens from the same tumors (Spearman r = 0.93; *P* < 0.0001; Fig 2D), demonstrating the accuracy of the IFA in measuring MET activation pharmacodynamics. Interestingly, the dose-dependent pharmacodynamic responses of the pY^1235^MET biomarker in the SNU-5 model corresponded to the dose-dependent tumor growth responses previously observed in another *MET*-amplified, MET–driven gastric carcinoma model—GTL-16—in a prior study [11], highlighting the ability of this assay to measure biologically relevant changes in MET activation.

**Fig 3:**
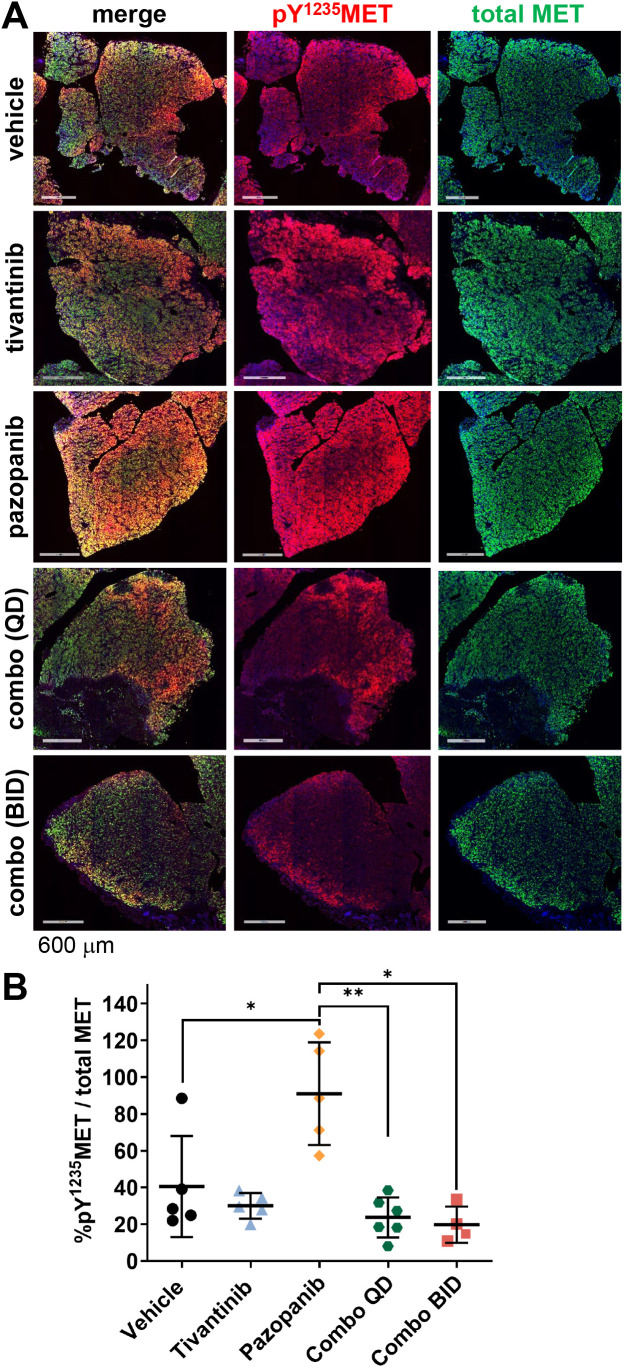
Fitness-for-purpose validation of IFA-based pY^1235^MET quantitation in sections of FFPE tumor specimens from SNU-5 xenograft models treated with pazopanib, tivantinib, or the pazopanib-tivantinib combination. Animals were treated for 8 days with vehicle (QD), tivantinib (200 mg/kg QD), pazopanib (100 mg/kg QD), or a combination of pazopanib (100 mg/kg QD) with either 200 mg/kg QD tivantinib or 200 mg/kg twice daily (BID) tivantinib (*n* = 4-6 animals per treatment group); tumor core biopsy specimens were collected 4 hours following administration of the final dose and flash frozen. **A,** Representative images of tumor tissue from each treatment group, showing staining for pY^1235^MET (red), total MET (green), and DAPI (blue). Total MET was detected using the AF488-conjugated anti–total MET D1C2 antibody. **B,** Multiplex IFA–based quantitation of the post-treatment pY^1235^MET/total MET ratio. Horizontal lines indicate the mean and standard deviation for each group, and asterisks indicate significant differences between the pazopanib-treated group and the vehicle- and combination-treated groups (*P < 0.05, **P < 0.01).

Fitness for purpose of the pY^1235^MET IFA was demonstrated in a SNU-5 tumor xenograft study designed to simulate as closely as possible a clinical trial application of the assay. Mice harboring SNU-5 tumors were treated with the VEGFR inhibitor pazopanib, the putative MET inhibitor tivantinib, or the pazopanib-tivantinib combination. VEGFR inhibition is known to yield compensatory increases in MET signaling [34], and our previously published phospho-MET ELISA results showed that 8 days of pazopanib treatment induced a significant increase in pY^1234/1235^MET/MET relative to vehicle treatment in the SNU-5 xenograft model, which was attenuated by cotreatment with the MET inhibitor tivantinib [11]. IFA analysis of the same SNU-5 tumors that were previously analyzed by ELISA demonstrated a similar trend: pazopanib treatment yielded a significant increase in the pY^1235^MET/MET ratio relative to vehicle treatment, while treatment with the pazopanib-tivantinib combination (with tivantinib administered either once daily or twice daily) yielded a significant decrease in pY^1235^MET/MET relative to single-agent pazopanib treatment (Fig 3A-B). These pazopanib- and combination-induced changes were likewise reflected in the absolute pY^1235^MET measurements, without normalization to total MET levels (Supplementary S2A-B Fig). Phospho-MET IFA analysis also demonstrated a similar pazopanib-induced increase in pY^1235^MET for another *MET*-amplified gastric carcinoma model, MKN45 (Supplementary S2C-D Fig).

### Application of the pY^1235^MET IFA to quantify subcellular localization of activated MET in the plasma membrane and nucleus in clinical and preclinical tumor specimens

**Fig 4:**
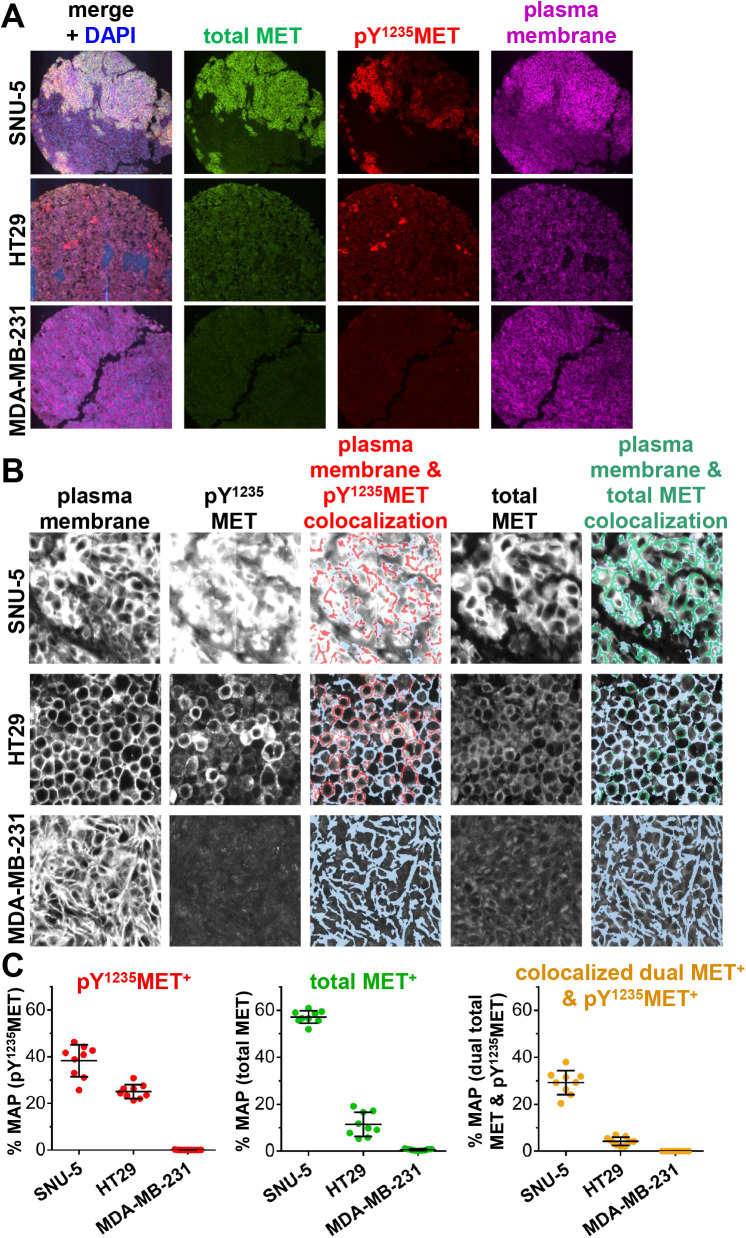
Quantitation of plasma membrane–localized pY^1235^MET and total MET in untreated tumor cell lines. **A,** Representative images of SNU-5, HT29, and MDA-MB-231 cell pellets stained with DAPI (blue) and for total MET (green), pY^1235^MET (red), and the plasma membrane marker Na^+^/K^+^-ATPase (magenta). Total MET was detected using the AF546-conjugated mouse monoclonal antibody MET4 (clone 8G6). **B,** High-magnification (20X) fluorescence images showing staining for plasma membrane marker Na^+^/K^+^-ATPase (left), pY^1235^MET, and total MET, as well as corresponding images showing masked regions of plasma membrane (light blue) with colocalized pY^1235^MET (red) or total MET (green), established using a custom Definiens algorithm. **C,** Quantitation of plasma membrane–localized pY^1235^MET (red), total MET (green) or colocalized dual pY^1235^MET and MET (orange) for each cell line, derived from plasma membrane–masked images. Mean percent membrane area–positive (% MAP) values (± standard deviations) are shown (*n* = 3 cell pellets per model and 3 image fields per cell pellet).

Given the biological importance of canonical MET signaling by plasma membrane-localized MET, we next sought to apply the pY^1235^MET IFA to the measurement of activated MET in the plasma membrane. To this end, we developed a multiplex IFA using antibodies to total MET, pY^1235^MET, and the plasma membrane marker Na^+^/K^+^-ATPase α1 subunit and simultaneously assessed the 3 analytes in cell pellets from the SNU-5, HT29, and MDA-MB-231 cell lines (Fig 4A). Definiens Tissue Studio Developer software was used to create a plasma membrane mask using the Na^+^/K^+^-ATPase marker (Fig 4B). The plasma membrane mask was then used to calculate the plasma membrane area and the percent membrane area positive (% MAP) for each of the other markers: total MET, pY^1235^MET, and total MET that is colocalized with pY^1235^MET (i.e., dual MET^+^ & pY^1235^MET^+^; Fig 4C). The % MAP values for total MET, pY^1235^MET, and colocalized dual MET + pY^1235^MET were highest in the SNU-5 cells, moderate in HT29 cells, and near 0 in MDA-MB-231 cells (Fig 4C), corresponding to the respective overall expression levels of these analytes across the different cell lines (as shown in Figs 1 and 2).

**Fig 5:**
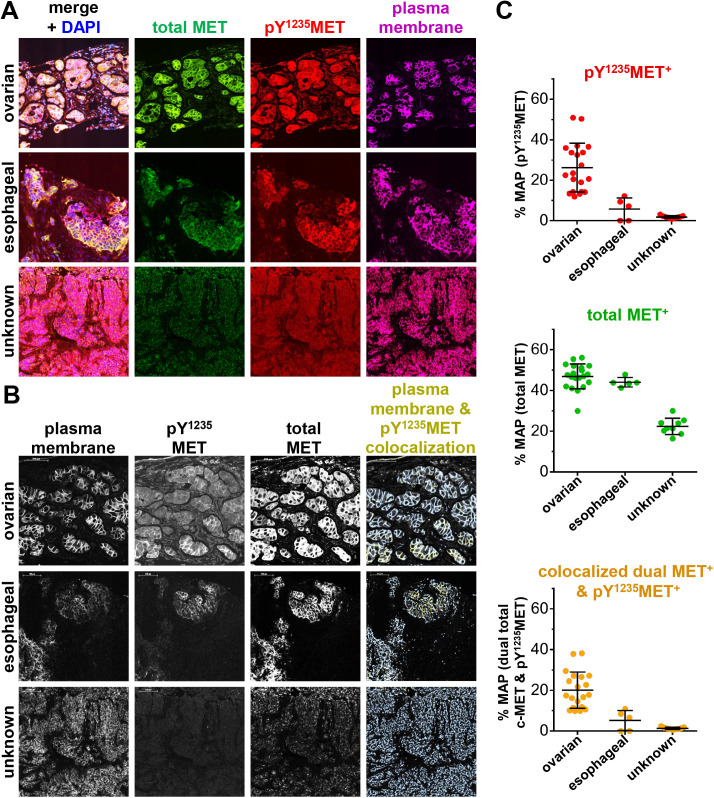
Quantitation of plasma membrane–localized pY^1235^MET and total MET in tumor biopsy specimens from untreated patients. **A,** Patient tumors stained with DAPI (blue) and for total MET (green), pY^1235^MET (red), and the plasma membrane marker Na^+^/K^+^-ATPase (magenta). Total MET was detected using the AF546-conjugated mouse monoclonal antibody MET4 (clone 8G6). Tumor histologies are noted; “unknown” indicates a patient with carcinoma of unknown primary origin. **B,** High-magnification fluorescence images showing staining for plasma membrane marker Na^+^/K^+^-ATPase (left), pY^1235^MET, and total MET, and corresponding masked images showing regions of plasma membrane–colocalized pY^1235^MET (gold) within the plasma membrane mask (light blue) established using a custom Definiens algorithm. **C,** Definiens-based quantitation of plasma membrane area positive for pY^1235^MET (top, red), total MET (middle, green), or colocalized, dual MET and pY^1235^MET (bottom, orange). Mean %MAP values are shown (*n* = 5-20 image fields per group).

To demonstrate the clinical suitability of the pY^1235^MET IFA incorporating a plasma membrane marker, we next measured levels of membrane-localized total MET and pY^1235^MET in pre-treatment core biopsy specimens obtained from patients with advanced carcinomas [12]. We measured high mean levels of plasma membrane-associated total MET in 2 patients with ovarian and esophageal carcinomas (46.9% MAP and 44.0% MAP, respectively), while a third patient with a tumor of unknown histology had a more modest level (22.3%; Fig 5A-C). The ovarian carcinoma case had a relatively higher mean % MAP pY^1235^MET level (26.2%) relative to those for the esophageal carcinoma (5.8%) and unknown histology (1.8%) cases. Accordingly, the % MAP values for colocalized dual MET & pY^1235^MET were highest in the ovarian carcinoma case (20.1%), followed by the esophageal and unknown histology cases (5.2% and 1.3%, respectively). Peptide blocking experiments confirmed the antigen specificity of the pY^1235^MET antibody’s staining of these clinical specimens (Supplementary S3A Fig). Together, these data confirm that this pY^1235^MET multiplex IFA can detect and quantitate differences in plasma membrane-localized total MET, pY^1235^MET, and colocalized dual MET & pY^1235^MET levels in clinical biopsy specimens.

**Fig 6:**
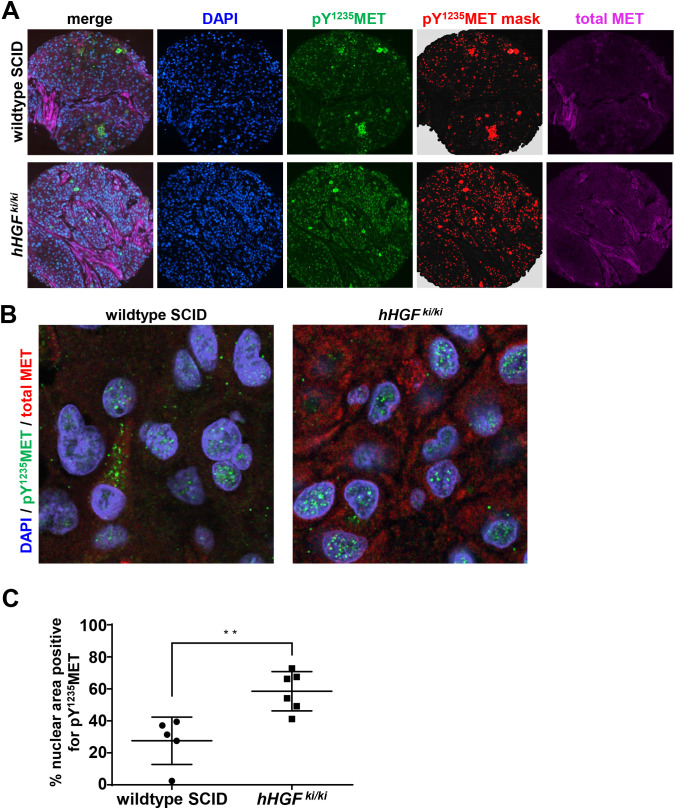
Constitutive HGF expression in H596 tumor–bearing, *hHGF* knock-in mice enhances nuclear pY^1235^MET levels in tumor cells. H596 NSCLC tumors were harvested from wildtype SCID or homozygous *hHGF* knock-in (*hHGF*^ki/ki^) mice 33 days after tumor implantation (via subcutaneous injection of tumor cells), and enhanced plasma hHGF levels in the tumor-bearing hHGF^ki/ki^ mice were detected, as described previously [29]. **A,** Representative immunofluorescence microscopy images and corresponding masked images showing pY^1235^MET expression in H596 tumors from wildtype SCID or *hHGF*^*ki/ki*^ animals. FFPE tissue sections were assessed by immunofluorescence microscopy after staining with DAPI (blue), AF546-conjugated anti-pY^1235^MET antibody (green), and AF647–conjugated MET4 anti-MET antibody (pink); Definiens-generated images of pY^1235^MET masks (red) are also shown. **B,** Representative high-magnification immunofluorescence confocal microscopy images of pY^1235^MET expression in H596 tumors from wildtype SCID or *hHGF*^*ki/ki*^ animals, showing nuclear (blue) localization of pY^1235^MET (green) and total MET (red). **C,** Quantitation of nuclear pY^1235^MET staining, expressed as the percent of DAPI-stained nuclear cross-sectional area positive (%NAP) for pY^1235^MET. Horizontal lines indicate means and standard deviations; each point represents a single animal (*n* = 5-6 animals per group).

**Fig 7:**
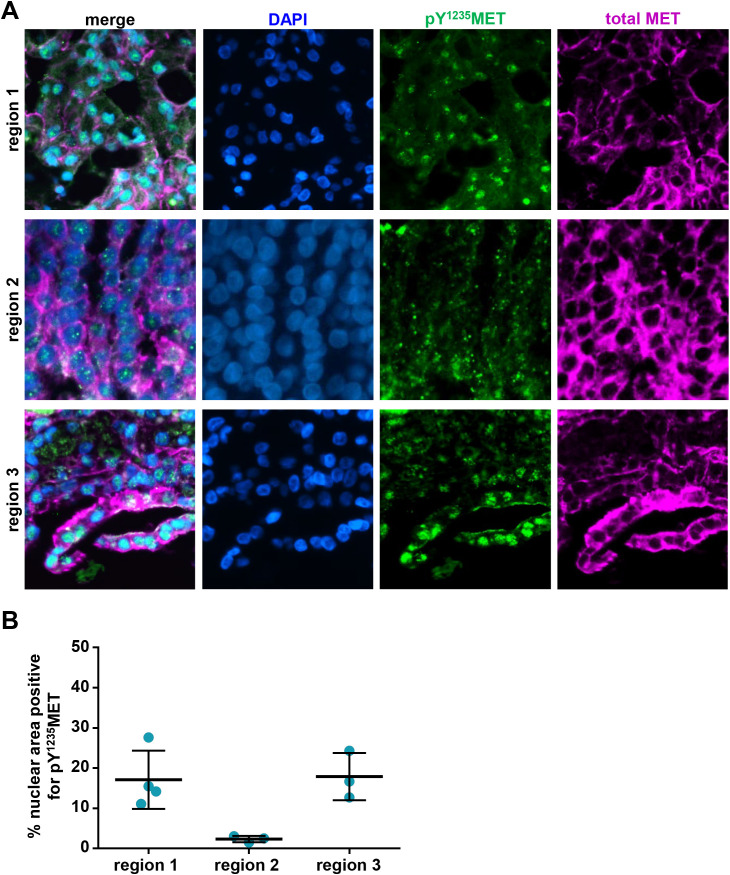
Detection and quantitation of nuclear pY^1235^MET in FFPE sections of hereditary papillary renal cell carcinoma tumor core biopsy specimens. **A,** Representative immunofluorescence microscopy images (40X magnification) of FFPE tumor biopsy sections stained with DAPI (blue) or antibodies to pY^1235^MET (green) or total MET (magenta), demonstrating the presence of nuclear-localized pY^1235^MET in human HPRC tumors. Total MET was detected using the AF647–conjugated MET4 anti-MET antibody. The 3 tumor regions shown represent 3 independent tumor biopsy cores sampled from the same lesion within a single patient. **B,** Quantitation of nuclear pY^1235^MET staining. Horizontal lines indicate means and standard deviations from analyses of 3-4 ROIs per region.

Nuclear localization of MET full-length protein and C-terminal fragments has been demonstrated to occur in a number of different tumor cell types and also in response to environmental changes such as decreased cell density and intracellular pH or increased HGF signaling [19,35]. To measure nuclear MET and pY^1235^MET using the multiplex IFA, we masked the nuclear area using the DAPI fluorescence signal and then quantified total MET and pY^1235^MET within that nuclear mask to calculate the respective percent nuclear area positive (% NAP) values. In a preclinical fitness-for-purpose study in NSCLC H596 tumor–bearing wildtype SCID or homozygous human *HGF* knock-in (*hHGF*^*ki/ki*^) mice [29], tumor cell nuclei from both wildtype SCID and *hHGF*^*ki/ki*^ mice were positive for pY^1235^MET as determined by IFA (Fig 6A-B). However, relative to wildtype SCID mice, H596 tumors from *hHGF*^*ki/ki*^ mice exhibited significantly higher % NAP levels of pY^1235^MET (31.4% vs. 60.2%; Fig 6C). Peptide blocking data confirmed pY^1235^MET antibody specificity for detection of nuclear activated MET (Supplementary S3B Fig). We also evaluated nuclear pY^1235^MET expression in 3 biopsy core specimens from a resected tumor nodule in a patient with hereditary papillary renal cell carcinoma (HPRC) and found that all 3 specimens were evaluable and positive for nuclear pY^1235^MET (Fig 7A-B). Within these specimens, many of the nuclei exhibiting strong pY^1235^MET staining did not appear to be positive for total MET staining using the C-terminus–directed D1C2 antibody, suggesting that the nuclear-localized pY^1235^MET fragment(s) lack the C-terminal epitope recognized by the D1C2 antibody (Fig 7A); these results are consistent with previous reports of a pro-apoptotic 40 kDa MET fragment (“p40MET”) that is generated by caspase cleavage and consists of MET residues 1000–1374 [36], thereby maintaining the MET NLS and Y1235 phosphorylation site while lacking the distal-most C-terminal residues that are presumably recognized by D1C2. Intratumoral heterogeneity was evident, with varying %NAP levels of pY^1235^MET across the 3 tumor regions: 17.1%, 2.4%, and 17.9%, respectively (Fig 7B). Together, these data indicate that this pY^1235^MET multiplex IFA can be used to detect and quantitate nuclear pY^1235^MET expression in clinical and preclinical tumor specimens.

### Tumor levels of pY^1235^MET and total MET in clinical specimens of non-small cell lung cancer and colorectal carcinoma

**Fig 8:**
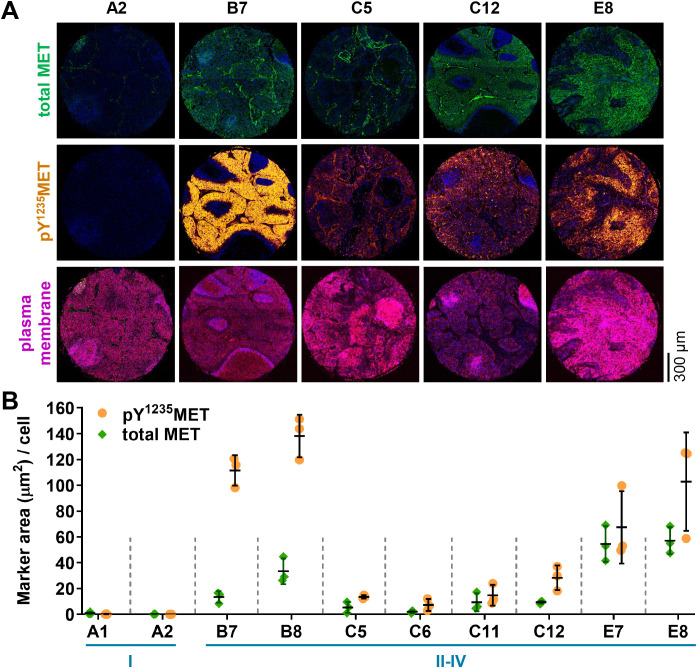
Levels of pY^1235^MET and total MET across tumors from patients with non-small cell lung cancer. **A,** Immunofluorescence microscopy images of representative FFPE sections of tumor core biopsy specimens stained for total MET (green), pY^1235^MET (gold), or Na^+^/K^+^-ATPase (magenta). Total MET was detected using the AF488-conjugated anti–total MET D1C2 antibody. Each column shows a single tumor specimen from a unique patient. **B,** Quantitation of marker area positive per cell for total MET and pY^1235^MET. Each x-axis label represents a unique tumor, and each of the following tumors were pairs collected from a unique patient: A1 and A2; B7 and B8; C5 and C6; C11 and C12; and E7 and E8. The disease stage for each specimen is noted in blue. Each point represents the value for an individual ROI (*n* = 3 ROIs per tumor); vertical lines indicate means ± standard deviations.

**Fig 9:**
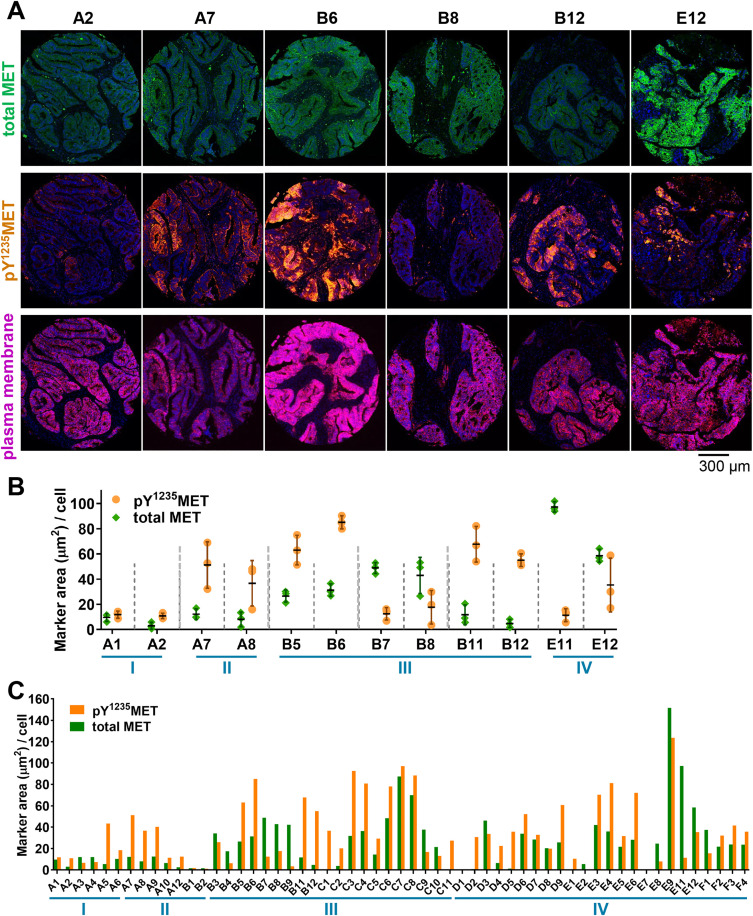
Levels of pY^1235^MET and total MET in tumors from patients with colorectal carcinoma. **A,** Images of representative FFPE tumor core biopsy sections evaluated by immunofluorescence microscopy for total MET (green), pY^1235^MET (gold), or Na^+^/K^+^—ATPase (magenta). Total MET was detected using the AF488-conjugated anti–total MET D1C2 antibody. Each column shows a single tumor specimen from a unique patient. **B,** Quantitation of marker area positive per cell for total MET and pY^1235^MET. Each x-axis label represents a unique tumor, and each of the following tumors were pairs collected from a unique patient: A1 and A2; A7 and A8; B5 and B6; B7 and B8; B11 and B12; and E11 and E12. The disease stage for each specimen is noted in blue. Each point represents the value for an individual ROI (*n* = 3 ROIs per tumor); vertical lines indicate means ± standard deviations. **C,** Mean marker area positive per cell for total MET and pY^1235^MET across all CRC tumor tissue microarray specimens.

*MET* genetic aberrations have demonstrated prognostic value in patients with NSCLC and colorectal carcinoma (CRC), and *MET* amplification and specific MET activating mutations are predictive markers for clinical response to MET-targeted therapies in these tumor types [37,38]. MET overexpression, however, has not been consistently predictive of response to such therapies across the multiple clinical studies that have employed diagnostic IHC assays to qualitatively assess MET expression [37–39]. Therefore, a quantitative clinical assay that directly measures MET activation levels, rather than measurement of total MET expression, may be more useful in identifying tumors that utilize MET signaling. As a first step toward the application of the pY^1235^MET multiplex IFA in understanding the relationship between total MET, pY^1235^MET, and response to MET-targeted agents, we assessed the prevalence and magnitude of MET and its activated pY^1235^ form in NSCLC and CRC cases in tissue microarrays. Total MET marker area in the 10 NSCLC patient tumors that were assessed did not always correspond to pY^1235^MET marker area within the same tumors; indeed, assay readouts of pY^1235^MET marker areas generally equaled or exceeded (in some cases, substantially) assay readouts of total MET areas, with mean pY^1235^MET and total MET levels ranging from 0.5–138.3 μm^2^/cell and 0.2–57.1 μm^2^/cell, respectively (Fig 8A-B). In the 57 CRC patient tumors examined, we also observed heterogeneity in the relative marker area/cell values for pY^1235^MET and total MET, with mean pY^1235^MET and total MET levels ranging from 0.3–123.6 μm^2^/cell and 0.01–151.7 μm^2^/cell, respectively (Fig 9A-C). While the marker area per cell for some specimens was higher for pY^1235^MET compared to total MET, the reverse was true for other specimens, and some specimens had similar marker area/cell values for both pY^1235^MET and total MET. In addition, tumor pY^1235^MET level was not associated with CRC disease stage (Fig 9B-C). As patient treatment and response information were not available for these tumor tissue microarray specimens, future analyses of total and pY^1235^MET levels in tumor biopsy tissue from patients treated with specific MET-targeting agents—and with accompanying baseline tumor molecular profiling and clinical response data—will be instrumental in defining the potential predictive value of pY^1235^MET IFA measurements in clinical studies.

## Discussion

Reliable, specific measurements of MET activation levels in tumor tissue—as an indicator of MET signal transduction—may facilitate better patient selection for MET-targeted oncology therapeutics, and the coupling of such measurements with detailed subcellular spatial information may provide clarity regarding the biological functions of various activated MET subpopulations within a tumor cell. The pY^1235^MET immunofluorescence microscopy assay described here yields this desired information while utilizing only a small number of tissue sections from a single tumor biopsy core, which, for previously validated, fit-for-purpose IFAs has been the number of biopsy sections that yields at least 3,000 viable tumor cells [40]. This IFA extends upon our previously developed tumor lysate–based sandwich immunoassay for pY^1234/1235^MET [11], which lacks the aforementioned subcellular localization information and also consumes an entire biopsy core. Importantly, the use in this IFA of an antibody that specifically binds the C-terminus of MET enables measurement of Y^1235^ phosphorylation in both full-length MET and the C-terminal cleavage products resulting from proteolysis during signal transduction—a feature unique to this IFA relative to the sandwich immunoassay, which measures only full-length MET. We have demonstrated here the specificity of our anti-pY^1235^MET antibody in microscopy-based measurements, as well as concordance between data from the pY^1235^MET IFA and the previously validated pY^1234/1235^MET sandwich immunoassay. The specimen collection and processing SOPs used for both this IFA and the previously published sandwich immunoassay specify minimal ischemia times (<5 minutes), which we have previously shown to be essential for preserving adequate pY^1235^MET (clone 23111) signal in the sandwich immunoassay [11] (S4-S5 Figs).

Because activated MET may be found both on the plasma membrane and in the nucleus, we also developed IFA panels to measure total MET and pY^1235^MET levels in both of these subcellular locations via image field segmentation using Na + /K + -ATPase and DAPI staining, respectively. The availability of these clinically fit-for-purpose assays for measuring specific subcellular populations of activated MET provides opportunities for broader analysis of MET biology in various tumor histologies, more thorough pharmacodynamic studies of the effects of MET signaling inhibition, and further understanding of how best to target MET signaling in different subcellular compartments using different classes of therapeutic agents. For example, quantitative measurements of total MET levels within the plasma membrane relative to other subcellular compartments may hold value as a predictive biomarker for response to MET-targeting antibody-drug conjugates such as telisotuzumab vedotin, which recently received accelerated FDA approval [39,41,42]. In contrast, for agents that perturb MET signaling—including MET TKIs and MET antagonist antibodies—global pY^1235^MET, which is indicative of overall MET activation levels, may be the optimum biomarker for both predictive diagnostic assays and for pharmacodynamic assays measuring small molecule− or antagonist antibody−induced attenuation of MET activation.

In addition, given the translocation of MET to the nucleus in response to changes in pH, oxidative stress, and HGF stimulation [8,16–20], as well as the associations between nuclear MET and EMT and therapeutic resistance [17,25], future studies demonstrating the breadth of nuclear MET expression in untreated patient tumors or in response to MET-targeting therapies may provide further insight into potential associations between MET nuclear localization and disease prognosis or therapeutic response. Indeed, several studies have examined the pro-apoptotic function of the p40MET intracellular MET fragment spanning residues 1000–1374, which contains the MET NLS and Y^1235^ phosphorylation site while lacking the extreme C-terminus and is found in the cytoplasm, nucleus, and mitochondria-associated endoplasmic reticulum membrane [36,43–45]. Our detection of a pY^1235^MET signal consistent with p40MET (i.e., containing Y^1235^ but lacking the MET distal C‑terminus) in the nuclei of tumor cells from HPRC patient specimens raises questions regarding the biological functions of various nuclear MET fragments and phospho-isoforms thereof in regulating tumor cell growth vs. tumor cell death—questions that may be addressed, in part, through future applications of the assays presented here. Importantly, while our assay measures modulation of nuclear pY^1235^MET levels, it does not distinguish between changes in nuclear translocation of full-length MET, nuclear translocation of cleaved MET fragments, or altered MET nuclear retention dynamics, thus underscoring the need for multiple experimental tools to fully explore questions of MET nuclear translocation dynamics.

To date, oncology therapeutics designed to inhibit MET signaling have yielded impressive activity only in patients that have been molecularly selected for specific MET aberrations, the most promising being *MET* exon 14–skipping mutations [1,2,5]. However, *MET* exon 14–skipping mutations do not always confer response to MET inhibitors, and two other common molecular selection criteria, *MET* gene amplification and MET protein overexpression, have often been insufficient for predicting response to MET-targeting agents [2,6,7]—suggesting a need for improved patient selection markers for this family of therapeutics. Our IFA analysis of total MET and pY^1235^MET levels across a panel of over 50 resected tumor specimens from patients with CRC or NSCLC demonstrate that total MET protein expression levels are not always associated with the levels of activated, pY^1235^MET, suggesting that quantitation of the latter may provide a more accurate measure of the level of MET signaling activity in a given tumor. We hypothesize that pY^1235^MET represents a nexus of the various pathways that can lead to high baseline MET activation—including *MET* amplification, MET overexpression, and *MET* exon 14-skipping mutations—and that high tumor levels of pY^1235^MET could be both necessary and sufficient for predicting response to MET inhibitors. However, it is possible that the presence of multiple oncogenic drivers in some patients could render pY^1235^MET measurements alone insufficient for fully predicting response.

This clinically validated, quantitative pY^1235^MET immunofluorescence assay represents a substantial step toward accurate, biologically meaningful measurements of tumor MET activation and its subcellular location. The results of our study indicate that tumor total MET levels do not always reflect activated (pY^1235^) MET levels, suggesting that independent assessment of pY^1235^MET as a predictive biomarker for determining response to MET targeted therapies is warranted in future clinical studies of MET-targeting agents, as is assessment of plasma membrane–associated MET as the accessible target for antibody-based targeted therapies.

## Supporting information

S1 Fig Specificity of pY^1235^MET detection following crizotinib treatment in A549 and GTL-16 cells. **A,** A549 or H596 cells were incubated with or without 100 nM crizotinib for 4 hours prior to stimulation with 20 ng/mL HGF for 15 minutes. Cells were harvested, fixed and permeabilized, and then stained with DAPI, rabbit anti-pY^1235^MET antibody (clone 23111), and Alexa Fluor (AF) 546-conjugated anti-rabbit secondary antibody. **B,** GTL-16 cells were treated with the indicated concentrations of crizotinib for 4 hours, flash-frozen, and then cell lysates were subjected to Western blot analysis using digoxigenin (DIG)-conjugated rabbit anti-pY^1235^MET antibody, an 800CW-conjugated anti-DIG secondary antibody (Licor), and AF488–conjugated anti-MET antibody (Cell Signaling Technology). Each blot was incubated overnight with the antibodies as well as either buffer or the unphosphorylated MET, pY^1234^MET, pY^1235^MET, or pY^1234-1235^MET peptides (present at a molar concentration 30 times that of each primary antibody). The pY^1235^MET signal is reduced by treatment with 100 nM crizotinib, and specific recognition of the pY^1235^ modification is demonstrated by the elimination of the pY^1235^MET signal by the pY^1235^MET and pY^1234-1235^MET peptides but not by the pY^1234^MET or unphosphorylated MET peptides.
(TIF)

S2 Fig IFA-based pY^1235^MET quantitation in FFPE tumor core biopsy sections from *MET*-amplified SNU-5 and MKN45 xenograft models treated with pazopanib, tivantinib or the pazopanib-tivantinib combination. Animals were treated for 8 days with vehicle (QD), tivantinib (200 mg/kg QD), pazopanib (100 mg/kg QD), or the combination of pazopanib (100 mg/kg QD) with either 200 mg/kg QD tivantinib or 200 mg/kg twice daily (BID) tivantinib (*n* = 4–6 animals per treatment group); core needle tumor biopsies were collected 4 hours following administration of the final dose and flash frozen. **A** and **C,** IFA-based pY^1235^MET measurements for each region of interest (ROI) from SNU-5 (A) or MKN45 (B) models treated with vehicle or pazopanib. Each point represents a single ROI. Bars indicate mean values, while horizontal lines indicate standard deviations and asterisks indicate significant differences between the pazopanib- and vehicle-treated groups (****P < 0.001). **B** and **D,** IFA–based quantitation of post-treatment tumor pY^1235^MET levels in SNU-5 (B) or MKN45 (D) models. Horizontal lines indicate the mean and standard deviation for each treatment group, and asterisks indicate significant differences between the pazopanib-treated group and the vehicle- and combination-treated groups (*P < 0.05, **P < 0.01).
(TIF)

S3 Fig Specificity of pY^1235^MET IFA for analysis of clinical and preclinical specimens is demonstrated by peptide blocking. **A,** Tumor tissue from the untreated esophageal carcinoma patient shown in Figure 5 was incubated with antibodies to C-terminal MET (D1C2), pY^1235^MET, and Na^+^/K^+^—ATPase, together with either buffer, a Y^1234^-phosphorylated MET peptide corresponding to amino acids 1229–1240 (“pY^1234^MET peptide”), or the same peptide phosphorylated instead at Y^1235^ (“pY^1235^MET peptide”). Each peptide was present at 20 times the molar concentration of the anti-pY^1235^MET antibody. Representative images show staining for DAPI (blue), total MET (green), pY^1235^MET (red), and the plasma membrane marker Na^+^/K^+^—ATPase (magenta). **B,** H596 tumor tissue from *hHGF*^ki/ki^ SCID xenograft models was incubated with anti-pY^1235^MET antibody together with either buffer or pY^1235^MET peptide (at 20X the molar concentration of the antibody); images show staining for DAPI (blue) and pY^1235^MET (green).
(TIF)

S4 Fig Uncropped, unadjusted Western blots for S1B Fig. Full blot images for S1B Fig are shown for pY^1235^MET (**A**) and total MET (**B**).
(TIF)

S5 Fig Raw data underlying all manuscript figures. Each spreadsheet tab contains the raw data corresponding to the specified figure panel.
(XLSX)
