# Supplementary figures and images for: Multiplex immunofluorescence microscopy assays for pharmacodynamic assessment of MET tyrosine kinase activation in the plasma membrane and nucleus
Source: PLoS One. 2026 May 12;21(5):e0349090. doi: 10.1371/journal.pone.0349090 (PMC13166943; doi:10.1371/journal.pone.0349090)

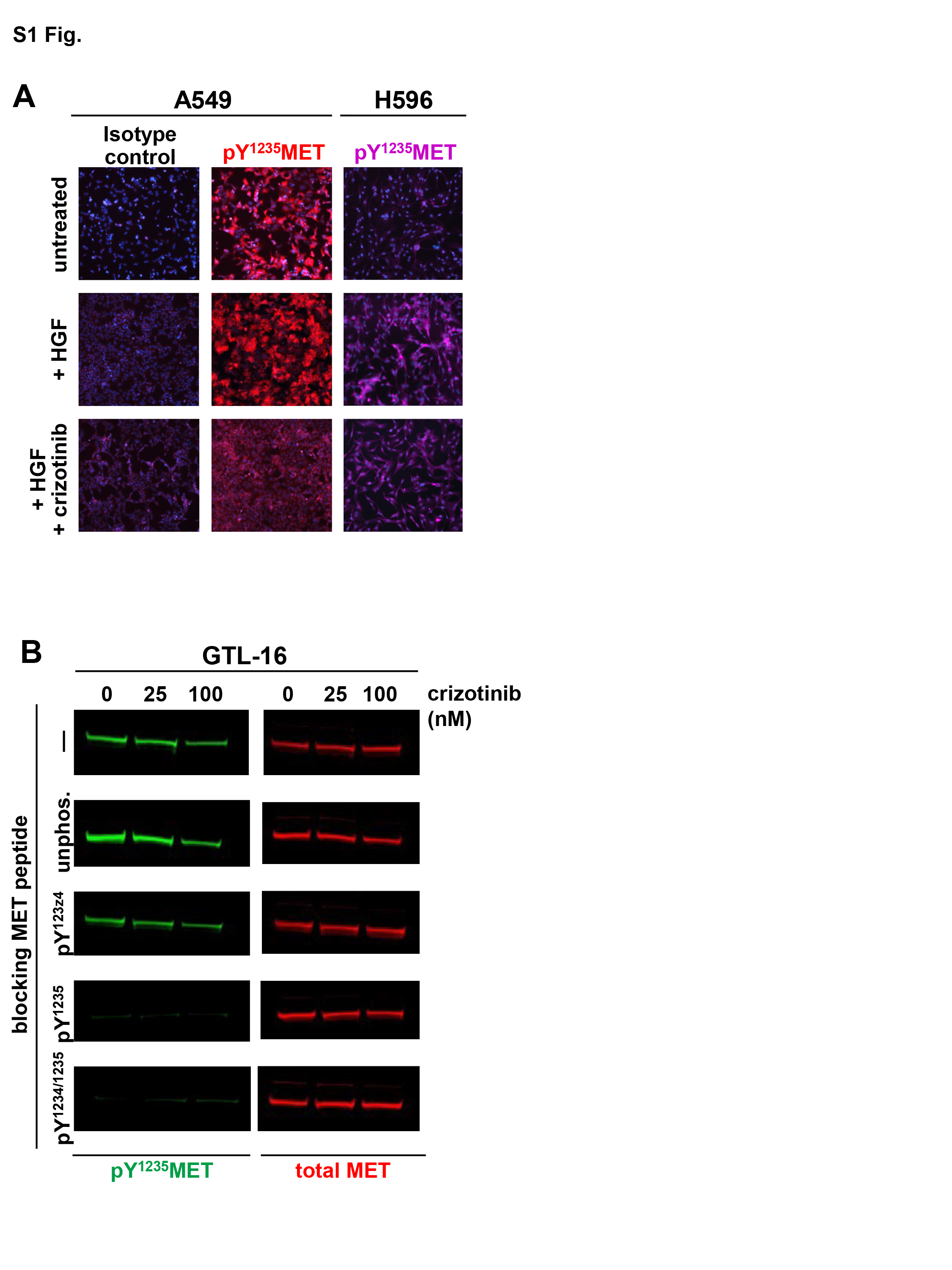

Supplement: S1 Fig — A, A549 or H596 cells were incubated with or without 100 nM crizotinib for 4 hours prior to stimulation with 20 ng/mL HGF for 15 minutes. Cells were harvested, fixed and permeabilized, and then stained with DAPI, rabbit anti-pY1235MET antibody (clone 23111), and Alexa Fluor (AF) 546-conjugated anti-rabbit secondary antibody. B, GTL-16 cells were treated with the indicated concentrations of crizotinib for 4 hours, flash-frozen, and then cell lysates were subjected to Western blot analysis using digoxigenin (DIG)-conjugated rabbit anti-pY1235MET antibody, an 800CW-conjugated anti-DIG secondary antibody (Licor), and AF488–conjugated anti-MET antibody (Cell Signaling Technology). Each blot was incubated overnight with the antibodies as well as either buffer or the unphosphorylated MET, pY1234MET, pY1235MET, or pY1234-1235MET peptides (present at a molar concentration 30 times that of each primary antibody). The pY1235MET signal is reduced by treatment with 100 nM crizotinib, and specific recognition of the pY1235 modification is demonstrated by the elimination of the pY1235MET signal by the pY1235MET and pY1234-1235MET peptides but not by the pY1234MET or unphosphorylated MET peptides. (TIF) [file pone.0349090.s001.tif]

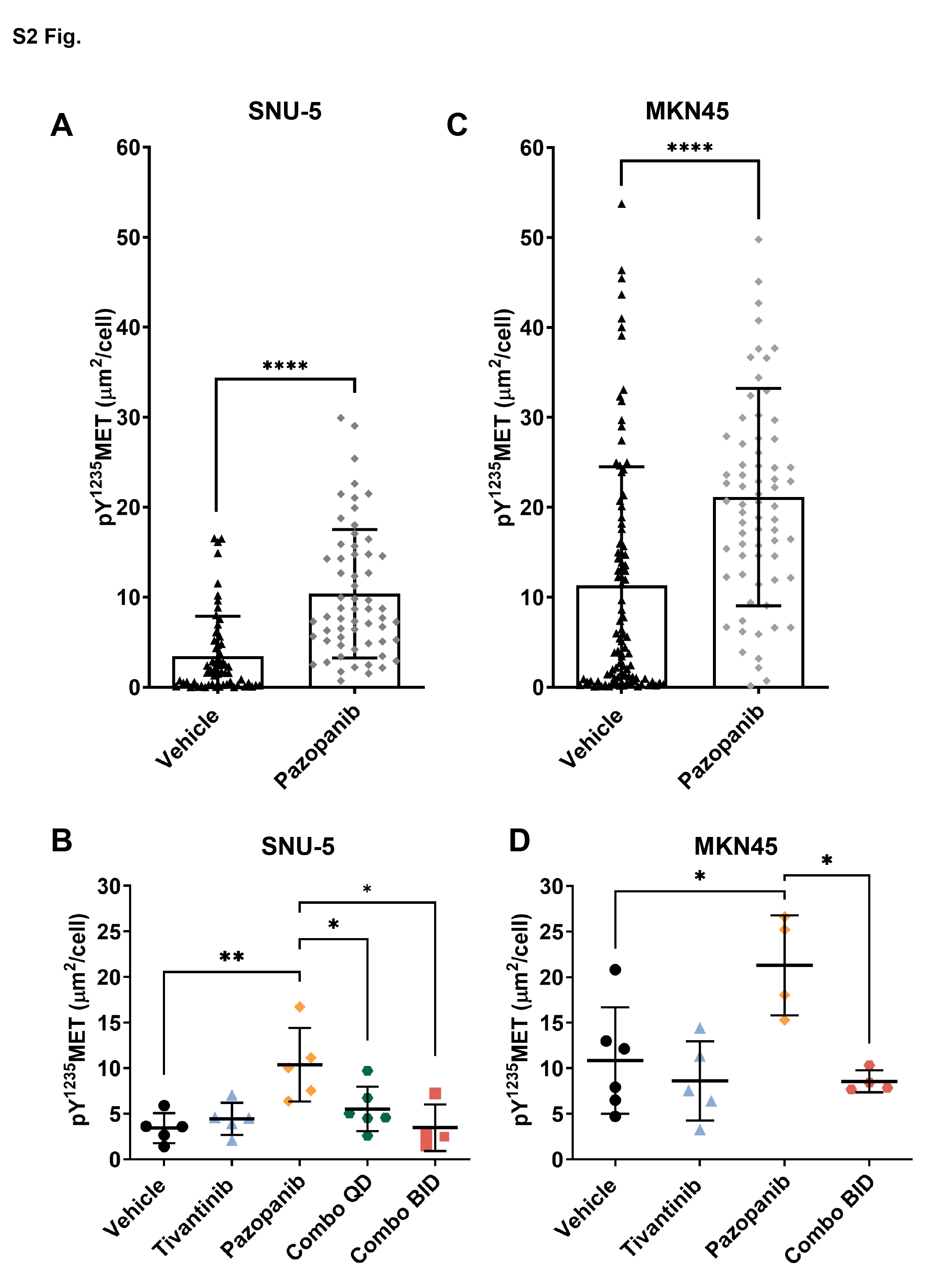

Supplement: S2 Fig — Animals were treated for 8 days with vehicle (QD), tivantinib (200 mg/kg QD), pazopanib (100 mg/kg QD), or the combination of pazopanib (100 mg/kg QD) with either 200 mg/kg QD tivantinib or 200 mg/kg twice daily (BID) tivantinib (n = 4–6 animals per treatment group); core needle tumor biopsies were collected 4 hours following administration of the final dose and flash frozen. A and C, IFA-based pY1235MET measurements for each region of interest (ROI) from SNU-5 (A) or MKN45 (B) models treated with vehicle or pazopanib. Each point represents a single ROI. Bars indicate mean values, while horizontal lines indicate standard deviations and asterisks indicate significant differences between the pazopanib- and vehicle-treated groups (****P < 0.001). B and D, IFA–based quantitation of post-treatment tumor pY1235MET levels in SNU-5 (B) or MKN45 (D) models. Horizontal lines indicate the mean and standard deviation for each treatment group, and asterisks indicate significant differences between the pazopanib-treated group and the vehicle- and combination-treated groups (*P < 0.05, **P < 0.01). (TIF) [file pone.0349090.s002.tif]

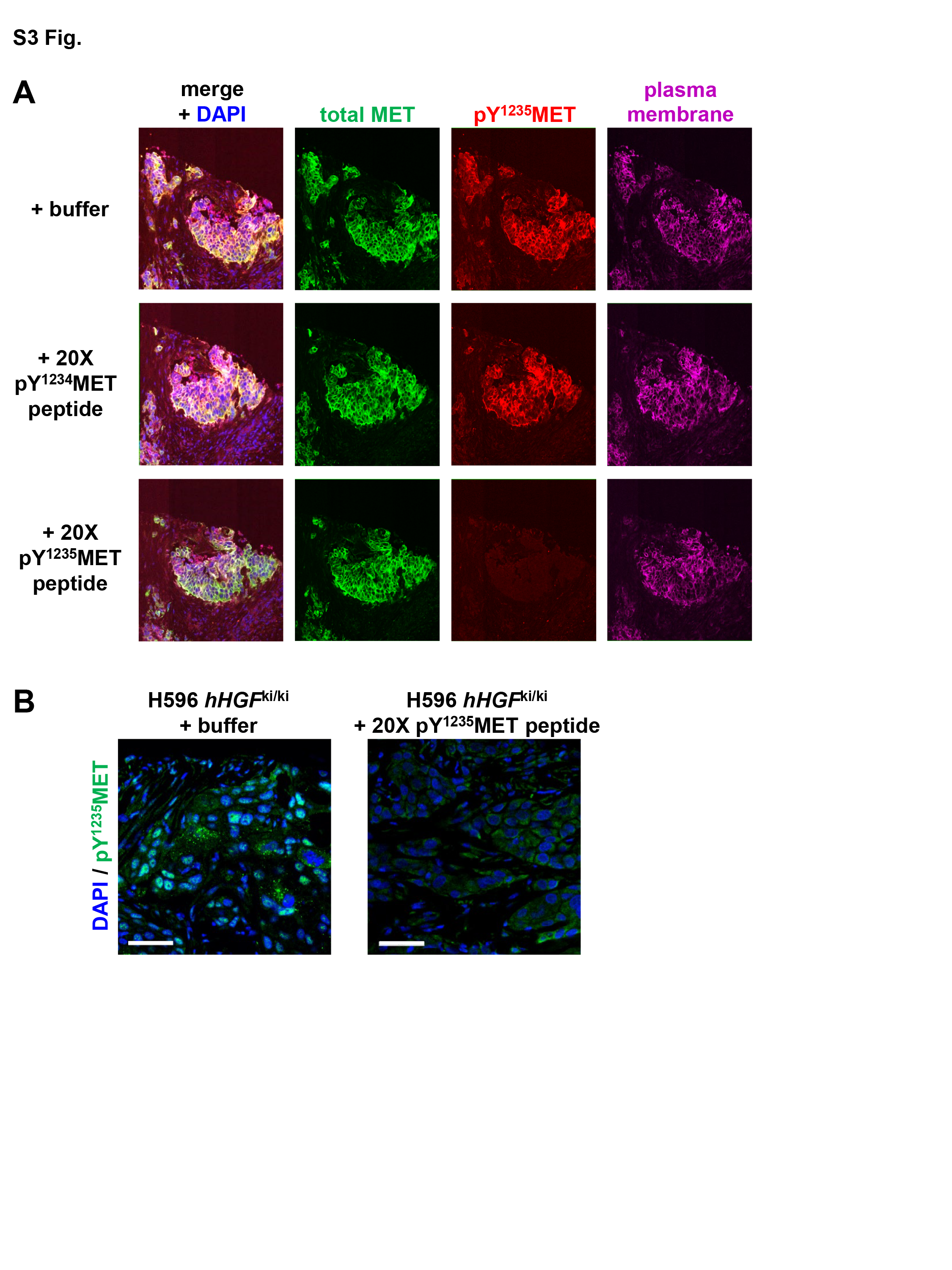

Supplement: S3 Fig — A, Tumor tissue from the untreated esophageal carcinoma patient shown in Figure 5 was incubated with antibodies to C-terminal MET (D1C2), pY1235MET, and Na+/K+—ATPase, together with either buffer, a Y1234-phosphorylated MET peptide corresponding to amino acids 1229–1240 (“pY1234MET peptide”), or the same peptide phosphorylated instead at Y1235 (“pY1235MET peptide”). Each peptide was present at 20 times the molar concentration of the anti-pY1235MET antibody. Representative images show staining for DAPI (blue), total MET (green), pY1235MET (red), and the plasma membrane marker Na+/K+—ATPase (magenta). B, H596 tumor tissue from hHGFki/ki SCID xenograft models was incubated with anti-pY1235MET antibody together with either buffer or pY1235MET peptide (at 20X the molar concentration of the antibody); images show staining for DAPI (blue) and pY1235MET (green). (TIF) [file pone.0349090.s003.tif]

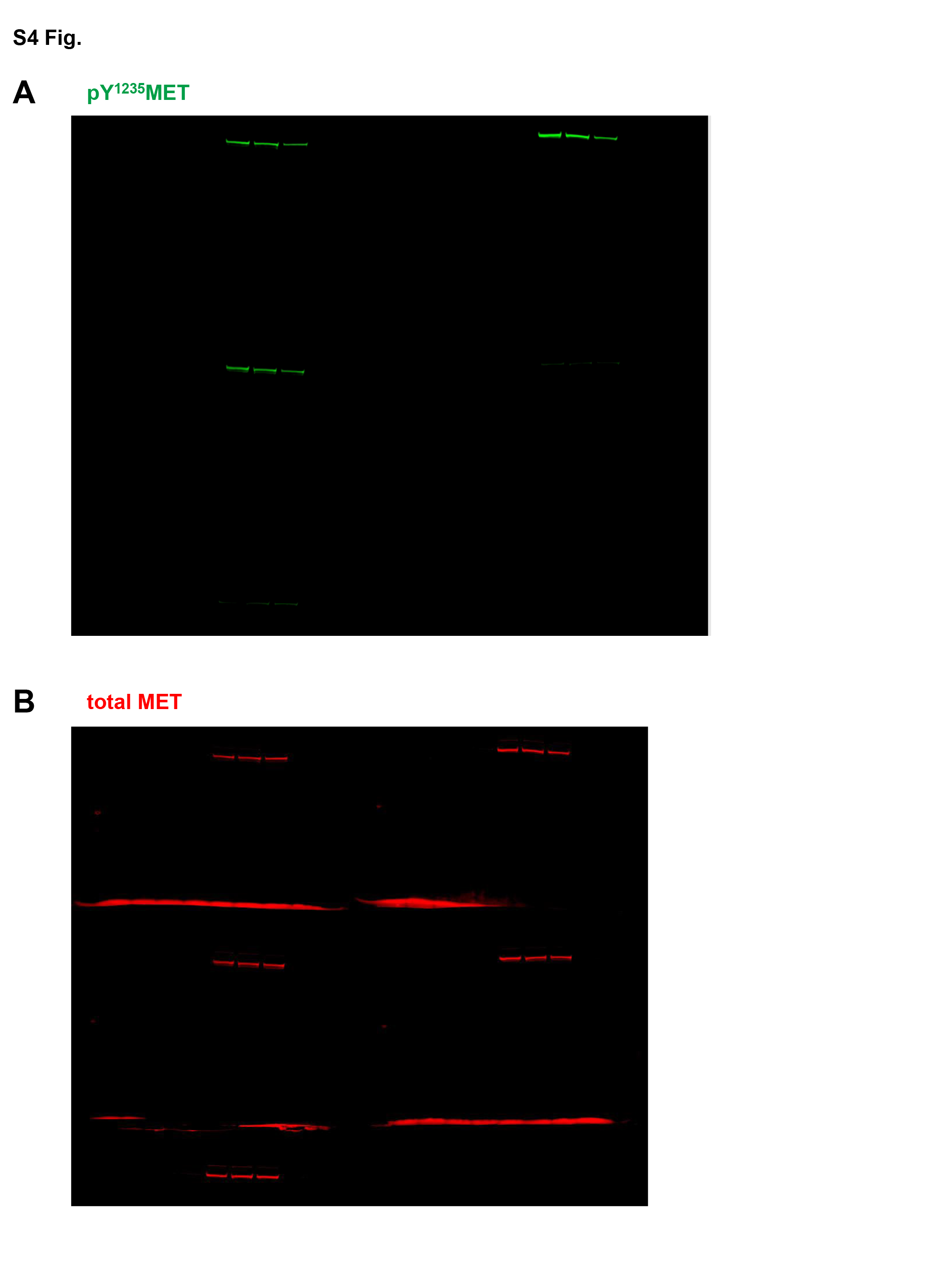

Supplement: S4 Fig — Full blot images for S1B Fig are shown for pY1235MET (A) and total MET (B). (TIF) [file pone.0349090.s004.tif]
